# Informal caregivers’ views on the quality of healthcare services provided to older patients aged 80 or more in the hospital and 30 days after discharge

**DOI:** 10.1186/s12877-020-1488-1

**Published:** 2020-03-12

**Authors:** Ingvild Lilleheie, Jonas Debesay, Asta Bye, Astrid Bergland

**Affiliations:** 1 Department of Physiotherapy, Faculty of Health Sciences, Oslo Metropolitan University, Oslo, Norway; 2 Department of Nursing and Health Promotion, Faculty of Health Sciences, Oslo Metropolitan University, Oslo, Norway; 3 Department of Nursing and Health Promotion, Faculty of Health Sciences, Oslo Metropolitan University, Oslo, Norway; 4 European Palliative Care Research Centre (PRC), Department of Oncology, Oslo University Hospital and Institute of Clinical Medicine, University of Oslo, Oslo, Norway; 5 Department of Physiotherapy, Faculty of Health Sciences, Oslo Metropolitan University, Oslo, Norway

**Keywords:** Healthcare, Informal care, Caregivers, Elderly, Quality of care, Patient-centered

## Abstract

**Background:**

In the European Union (EU), informal caregivers provide 60% of all care. Informal caregiving ranges from assistance with daily activities and provision of direct care to helping care recipients to navigate within complex healthcare and social services systems. While recent caregiver surveys document the impact of informal caregivers, systematic reviews show that they have unmet needs.

Because of the political desire to reduce the length of hospital stays, older patients are discharged from the hospital ‘quicker and sicker’ than before. The transition between different levels of the healthcare system and the period after hospital discharge is critical for elderly patients.

Caregivers’ perspectives on the quality of older patients’ care journeys between levels of the healthcare system may provide valuable information for healthcare providers and policymakers. This study aims to explore older patient’s informal caregivers’ views on healthcare quality in the hospital and in the first 30 days after hospitalisation.

**Method:**

We conducted semi-structured individual interviews with 12 participants to explore and describe informal caregivers’ subjective experiences of providing care to older relatives. The interviews were then transcribed and analysed thematically.

**Results:**

The analysis yielded the overarching theme ‘Informal caregivers – a health service alliance – quality contributor’, which was divided into four main themes: ‘Fast in, fast out’, ‘Scant information’, ‘Disclaimer of responsibility’ and ‘A struggle to secure professional care’. The healthcare system seemed to pay little attention to ensuring mutual understandings between those involved in discharge, treatment and coordination. The participants experienced that the healthcare providers’ main focus was on the patients’ diseases, although the health services are supposed to view patients holistically.

**Conclusion:**

Based on the information given by informal caregivers, health services must take into account each person’s needs and preferences. To deliver quality healthcare, better coordination between inter-professional care teams and the persons they serve is necessary. Health professionals must strengthen the involvement of caregivers in transitions between care and healthcare. Future work should evaluate targeted strategies for formal caregivers to cooperate, support and empower family members as informal caregivers.

## Background

In the EU, 60% of all care is provided by informal caregivers, defined as individuals who have a significant personal relationship with and provide a broad range of unpaid assistance to an older person or an adult with a chronic or disabling condition outside of a professional or formal framework [1]. The contribution of these caregivers constitutes a great resource for society, and within the Organization for Economic Co-operation and Development (OECD) countries, this care has been calculated to exceed the expenditure on formal care [1, 2]. Informal caregiving ranges from assistance with daily activities and provision of direct care to helping the care recipient to navigate within complex healthcare and social services systems [3]. In general, informal caregiving is more intensive, complex and longer lasting than in the past, and caregivers rarely receive adequate preparation for their role [3]. Recent caregiver surveys document the impact of informal caregivers [4, 5], but systematic reviews of the international literature reveal that informal caregivers have several continuously unmet needs [6, 7]. The quality of healthcare is of great importance to informal caregivers as well as the older patients, and the quality of health services for older people has an impact on the assistance needed from their caregivers.

The World Health Organisation (WHO) defines quality healthcare as care that is effective, efficient, integrated, patient centred, equitable and safe [8]. Additionally, the degree to which healthcare quality can be defined as acceptable is strongly dependent on service providers’ ability to meet the needs of the users [9] and adapt to their expectations and perceptions. The patients’ interests do not always coincide with those of their relatives [10], and family and informal caregivers may perceive the situation differently than the patients [11].

The overt political aim of reducing the length of stays in somatic hospitals has been explicit in several countries for years [12, 13]. As the average length of stay declines, elderly people are being discharged from the hospital ‘quicker and sicker’ than before [14–16]. The transition between the healthcare system and the period subsequent to hospital discharge is critical for elderly patients [17, 18]. Indeed, readmissions within 30 days after hospital discharge, referred to as ‘30-day rehospitalisations’, are frequent adverse outcomes for this population [19, 20] and have deleterious consequences, including loss of autonomy, increased mortality [21, 22] and high socioeconomic costs [23, 24]. The role of informal caregivers in the care of older patients following hospital discharge is described and recognised as important but remains understudied [3]. Little research has been carried out on how informal caregivers perceive the discharge of older family members from hospital to their own home and the first month after hospitalisation [25]. The caregivers’ perspectives on the older patients’ care journeys between levels of the healthcare system may therefore provide valuable information to guide quality improvements in health services [26]. Thus, this study aims to explore older patient’s informal caregivers’ views on the quality of health services in hospitals and the first 30 days after hospitalisation. Through the lens of healthcare quality, we hope to provide useful information to reduce the gap between policy and healthcare for older people. Furthermore, our paper might be of importance when tailoring education for informal caregivers as well as for those providing health services for older patients. In addition, the study could add important knowledge about how to facilitate informal caregivers’ participation in health services and provide a broader understanding of older patients’ and caregivers’ current situation.

## Methods

This study was part of a larger project addressing cross-sectoral care transitions for older patients from specialised hospital care to municipal health and care services, including the first 30 days after discharge [27].

### Design

The study used a qualitative descriptive approach because we desired a straightforward description of the phenomenon of interest. This approach is very useful when researchers want to know, regarding events, who were involved, what was involved, and where did things take place [28]. We intendes to capture the informal caregivers experiences to the extent that was possible. Thus, there was no pre-selection of study variables, no manipulation of variables, and no prior commitment to any one theoretical view of a target phenomenon. The goal was to obtain cases deemed rich in information for the purpose of saturating the data [28].

We sought to understand the complexity of the patients’ and informal caregivers’ situations through the perspectives of the informal caregivers’ experiences and carried out individual semi-structured interviews with open-ended questions to capture the way in which they experienced the healthcare services delivered to older patients in hospital and 30 days after discharge.

In addition, we followed the consolidated criteria for reporting qualitative studies (COREQ) [29].

### Setting and participants

In Norway, healthcare is mainly state funded and accessible to all inhabitants. Hospitals are primarily responsible for acute and specialised healthcare services, and the primary healthcare services in the municipalities provide healthcare suitable for early identification and follow up of somatic and mental health issues [30]. Home care services in Norway are administered by the municipality for people who require healthcare services for long or short periods as a result of illness, impaired health, old age or other factors [31].

We recruited a purposive sample of participants [32] from the relatives of patients admitted to an acute geriatric ward at a large hospital in Norway. The old patients were multi morbid, aged from 82 to 97 years old, living at home prior to hospitalization and were transferred to one of the following services: short-term stay at a nursing home (intermediate care) or own home with health services from the municipality. Fourteen informal caregivers were asked to participate and twelve agreed to be interviewed approximately 30 days after the patients were discharged from hospital. Purposeful sampling means selecting individuals that are especially knowledgeable about or experienced with a phenomenon of interest, which contributes to answering research questions [33].

Acute geriatric wards are defined as wards with an independent physical location and structure that are run by a specialised multidisciplinary team with direct responsibility for providing care for older people with acute medical disorders [34]. The inclusion criteria were that the informal caregivers’ relatives had to be aged 80 years or above, living at home prior to hospitalisation and were to be transferred to one of the following services: short-term stay at a nursing home (intermediate care) or own home, with health services provided by the municipality. Twelve informal caregivers agreed to participate and were interviewed approximately 30 days after the patients had been discharged from the hospital.

Participants ranged in age from 42 to 79 years old. The relationship to the patient varied from sons and daughters to friends and neighbours.

### Data collection

Data collection was carried out between September 2017 and March 2018. A semi-structured interview guide with open-ended questions was developed. The questions were based on established standards for quality in healthcare [8] (see Table 1). The interviews were conducted with caregivers one on one approximately 30 days after the patient they were caring for was discharged from the hospital. The interviews lasted from 30 to 105 min and were performed by the first author, who is an experienced physical therapist with long experience working with elderly patients and their relatives. All interviews were audiotaped and transcribed verbatim by a professional transcriber. Participants’ names and other personal identifiers were removed from the transcripts.

**Table 1:** Interview guide

1	Could you please describe how you experience the patients’ situation right now?
2	Please describe the hospital stay as you experienced it.
3	Please describe the discharge process as you experienced it.
4	Please describe how you experienced the transfer between the hospital and the healthcare services in the municipality.
5	Please describe how you experienced the cooperation/coordination between the personnel at the hospital and those in the healthcare services in municipality care.
6	Please describe how you experienced the patients’ situation after discharge.
7	Please describe whether the care professionals at the hospital and in the municipality have included you in the healthcare process.
8	Looking back, is there anything you think should have been done differently regarding the healthcare the patient has received?

Regarding the number of participants in qualitative studies, Creswell and Poth [32] recommend between five and twenty-five interviews, while Morse [35] suggests at least six.

### Data analysis

The interviews were audio recorded and transcribed verbatim. We analysed the data using thematic analysis [36, 37], a method for identifying, analysing and reporting patterns in qualitative data. Initially, all the authors read the transcribed material in an open way, searching for meaning and patterns. We used the analytical approach described by Braun and Clarke [36] to ensure consistency in the analysis of the data. This approach has been documented as robust across a wide range of disciplines [38]. To maximize reliability and secure validity we used the criteria for “trustworthiness” outlined by Lincoln and Guba (1985) [39]. The criterion of credibility was satisfied through open-ended questioning, loyalty to the data material and by giving a thorough description of the methods used. We met the transferability criterion by presenting comprehensive and thorough descriptions of the data through quotes from participants. The criterion for reliability was met by repeated reading of the transcripts by two of the authors (I. L. and A. B.). The interesting features of the data was identified and transformed into a set of codes. These codes were then categorised into potential themes. The themes were talked through and reflected on by all authors to ensure their relevance to the research questions [40]. All the authors who performed the analysis were educated in the health fields of physiotherapy (two), nursing (one) or nutrition (one) and have extensive research and/or clinical experience in the field of elder healthcare. The themes were then refined to ensure that each was meaningful and clear but distinct from other themes [41]. See Table 2 for an example of the coding strategy.

**Table 2:** Examples of the coding strategy

Unit meanings	Initial codes	Themes
*When I talked with them at the hospital, we got good information about where she was or something like that; they had told me that she wasn’t going home until they found out what it was, and that I thought was absolutely amazing – I have never met a doctor in the hospital who has told me that they would find out what mattered with the person before the person is sent home. Neither did my daughter, so I asked her several times, “Have you heard correctly? Did they really say that?’ But they didn’t, they sent her home before she had been fully investigated … Of course, they may not find it out; of course, it can be acute; perhaps something that will get better without intervention; but when it is the heart and they cannot complete a cardiac examination, I think that is unsatisfactory. And then she was sent home … They called from the hospital and talked to me as her relative about it, so then I said, especially in response to home nursing and home care, that clearly I cannot take responsibility, I have not recovered from having had cancer, I am very worn out, so you have to fix it. She must have accommodation elsewhere, and transport and everything you need to arrange. You cannot rely on relatives to handle this, and have no protests about it, so they arranged a wheelchair taxi and mom came home at nine in the evening and the home service carer arrived. She may have told you that*.	Discharge Information Diagnostication Expectation Efficiency Holistic approach Biomedical approach Expectations about holistic approach Caregiver burden Blurred responsibility Transportation	**Fast in, fast out – the tension between appropriate versus efficient healthcare**

### Ethical considerations

The study was preapproved and registered by the Norwegian Centre for Research Data (No. 53110). The first author asked the older patients for the name of their informal caregivers as well as for permission to contact and interview them about the patients’ situations. The participants were provided with verbal and written information outlining the purpose of the study. We informed them that participation was voluntary and about their right to withdraw from the study at any stage. We assured them that this would not affect the patients’ current or future access to services. We obtained written and informed consent from all participants and guaranteed their confidentiality.

## Results

The analysis yielded four main themes, which are included in the overarching theme ‘Informal caregivers – a health service alliance – quality contributor’. This overarching theme verified the importance and quality-contributing role of caregivers as ‘knowledge keepers’ about patients, who activate health service resources to manage the situation of the older patient. The caregivers indicated that the quality of the health service depends on the tension between appropriateness and efficiency, the mutual exchange of information and understanding as well as the relevant competence of the formal care providers in managing the complexity of the older patients’ health services. The four main themes, along with selected quotations from the interviews to illustrate the findings, are presented in the following four sections.

### I: fast in, fast out – the tension between appropriate and efficient healthcare

The informal caregivers said that the hospital stays were characterised by treatment based on a biomedical approach, with little focus on the *patient’s preferences and the complex needs of older patients.* Furthermore, for short stays, very small or no changes in the older patients’ complex health status were reported. The average hospitalization time for the patients were 7 days (minimum 5 to maximum 15 days). In our study, the feelings according discharge did not differ from symptoms or former experiences of hospitalizations. Instead, several of the informal caregivers experienced that the main concern for the health services was to be efficient – in the sense of getting the patients examined and discharged as rapidly as possible – in order to save money. The participants said that as caregivers they expected rapid discharge based on the financial situation of the healthcare system. They understood the necessity to *free up beds* but questioned whether the patient’s return to home happened without an assessment of whether it was reasonable or safe for the patient, or whether the patient would receive satisfying follow-up from the healthcare services in the municipality. The informal caregivers indicated that they mostly were satisfied with the patients’ hospital stays. They explained their opinion of the discharge in this way:

*When they had found nothing, or nothing they could explain medically, then I think it is pretty OK to decide to discharge because they would rather make room for sick patients – someone who really needs it. They had taken X-rays and examined the brain*. (C2)

Nevertheless, several caregivers pointed out that the discharge occurred earlier than expected:

*When I talked with them at the hospital, we got good information about where she was or something like that; they had told me that she wasn’t going home until they found out what it was, and that I thought was absolutely amazing – I have never met a doctor in the hospital who has told me that they would find out what mattered with the person before the person is sent home. Neither did my daughter, so I asked her several times, ‘Have you heard correctly? Did they really say that?’ But they didn’t; they sent her home before she had been fully investigated … Of course, they may not find it out; of course, it can be acute; perhaps something that will get better without intervention; but when it is the heart and they cannot complete a cardiac examination, I think that is unsatisfactory. And then she was sent home … They called from the hospital and talked to me as her relative about it, so then I said, especially in response to home nursing and home care, that clearly I cannot take responsibility, I have not recovered from having had cancer. I am very worn out, so you have to fix it. She must have accommodation elsewhere, and transport and everything you need to arrange. You cannot rely on relatives to handle this, and have no protests about it, so they arranged a wheelchair taxi and mom came home at nine in the evening and the home service carer arrived. She may have told you that*. (C5)

Another caregiver stated that her grandmother’s care had essentially ended with the treatment that the hospital could do, but she would need follow-up care from the district:

*I do understand that when they can’t do anything more based on their resources. It’s not right to be in the hospital. I understand that. The hospital has not sent her out too early in terms of what they could do there; it is more that the district would rather just send her home and say that home nursing can come by. I think the city district is more concerned about money and economy.* (C23)

All the caregivers found that they had become the focus of biomedical approaches. One made the following observation:

*It’s going to be like this: ‘Now we have arranged the pills, and the situation with the pills is OK and stabilised, so now you can go home, because we must have your bed for someone else.* (C6).

The caregivers stated that healthcare in the hospital was not tailored towards the individual person’s needs, which is in fact essential for high-quality care and is increasingly recognised as being associated with improved health. They experienced that the healthcare personnel did not practice holistic healthcare that viewed the person behind the disease, considering that person’s perspective and treating him or her as a unique individual. One caregiver missed the staff’s more focused action for the rehabilitation of his mother:

*There was no action, as I perceived it, to get her out of bed or to try to get her back on her feet and enhance her functionality. It was kind of okay that she was in bed and just wanted to be there. I felt as if nothing else was happening except that she was able to do what she wanted, in a way, and that was to stay calm, by herself.* (C9)

Another stated:

*Nothing has really changed from before she was in the hospital... the only difference now is that she gets a little closer follow-up from the district and that they have become more attentive to her, in a way. First, she was offered a stove guard, and then she was given this walking chair, which she is very pleased with. So, it’s really a big difference since she was in the hospital. She got to learn from a physiotherapist in the hospital how to use it, so now she is very happy with it, and can get out and go for a walk. She has become a lot more mobile … it’s really the best thing that has come out of her stay in hospital*. (C8)

During the hospital stay and in the discharge process, the participants reported that the healthcare personnel were constrained by systemic determinants that made it difficult for them to consider what mattered to the patient or the informal caregiver:

*We talked to the nurse who had responsibility for my mother and told her that she must not be discharged until she’s better. Instead of having her for a week, if they only had her for a few days, then they might get her back like that time and again. I said I think it’s a bit shameful that you send her out before she’s better. They seemed to listen to what I said, but I had the impression that those higher up in the system don’t listen to that.* (C1)

In cases where patients had difficulties coping with everyday life after discharge, they were transferred to an intermediate care unit in the district. The informal caregivers had trouble understanding the purpose of these stays. The participants described intermediate care units as follows:

… *a waiting room with too few resources to be able to meet the need of the patients.* (C6)

*It [a short-term stay] is probably just a stopover to borrow some time. The staffing is too low. They don’t have time to take care of every single patient*. (C2)

The informal caregivers saw these units as a storage place for the patients until their health situation had recovered to a necessary minimum for them to manage in their own homes. The informal caregivers did not feel the needs of the patients were being addressed properly in the integrated care units. This caused them to ask for more coordination between the different levels in the healthcare system:

*In the transition there should have been something more.* (C13)

All the caregivers described the hospital stay as pleasant and safe, as the following examples illustrate:

*There was nothing to say about it. They were gentle, nice carers. The doctor came soon after the patient arrived and examined her and talked to her, and, yes, they did that!* (C2)

*My mother (a former nurse), enjoyed being in hospital. She’s pretty funny then, my mother. She liked her stay in hospital well because there were so many nice people. She is very positive and … really very social. She is very outgoing, and she gets her energy by being social and talking to people. So, it happened that people came in all the time and had to ask her about things, and there was even a doctor who took his exam with her as a patient, so she thought this was a great thing. … But then after five days, she wanted to go home. Then she thought it was nice to come home. Because it was quite tiring for her, in a way, to relate to all these different people all the time.* (C8)

### II: scant information – the tension between short-term versus long-term goals and openness versus closeness

Caregivers played a crucial role in providing information during patient hospital stays and after discharge. Several of the participants mentioned information as a critical aspect of good quality in healthcare. This included both the aspect of healthcare professionals providing necessary information, and the aspect of them themselves searching and receiving sufficient information to being able to follow up the patient’s needs in a satisfactory manner. Many of them reported that insufficient information was provided by health care professionals about the coordination within the health service systems, which services the older patient would receive and when they would be received and the persons that would be involved. They missed information by not searching for it, as they found it difficult to know what to ask. They found the situation complicated. The participants reported therefore that they had inadequate involvement in the planning of discharge and care. While some of the respondents experienced satisfactory communication and information from the healthcare service, others reported that scant information that left them with few possibilities to participate in or contribute to the process with their knowledge:

*The system is not self-explanatory and I didn’t really know anything, what to ask for and so forth. They should have given her better information. Relatives and patients need better information* (C5).

They reported that the health services in the district were typically aware of the discharge from the hospital, but plans for a satisfactory transfer and follow-up were not always in place. Family caregivers, though valuable, were often not involved before decisions were made, and the health workers did not reflect on how their decisions affected the situations or might lead to extra stress for the informal carers:

*There wasn’t a network meeting; they just sent him straight home. We did not know who the nurse contact was, or who was his primary contact. Totally no information! Who should take care of him? It was me, that was all. I was exhausted myself.* (C11)

Most of the information the informal caregivers received came from the patient. Due to the patients’ impaired health condition, this information was sometimes perceived as inadequate and incomplete. In such cases, the caregivers had to take responsibility for getting the necessary information to ensure a secure follow-up of the patient after discharge. One participant emphasised the need to ensure that relatives are better informed:

*If we are not there to ask, we will not get any information.* (C6)

When the informal caregivers lacked sufficient information and were left with unanswered questions, they did not know whom to contact:

*Maybe I can call and talk to the client’s office? But is it just to make contact? I don’t know, either; I’ve never been in anything like that.* (C3)

The informal caregivers were usually not aware of which or whether any information had been transferred from the hospital to the municipal health service, and this resulted in uncertainties and worries:

*I’ve been worried about that. In fact, I have not been sure about the communication between the hospital and the home service – whether it has been any good.* (C5)

In addition, they did not know what help the patients could expect or what they were entitled to from the primary care services:

*We suddenly became uncertain whether we had to apply for home nursing and things like that. We were given a stack of papers, so it seemed as if my mother and I had to apply, but then it turned out that this was already in place, that she had rights and that things were arranged. But we had not brought it with us*. (C9)

The informal caregivers described some of the most successful transitions to occur in cases were an interdisciplinary team from primary care offered health services to the patients following discharge. They reported that these teams offered a process involving two or more health professionals with complementary backgrounds and skills with a focus on assessing, planning or evaluating patient care. Moreover, the informal caregivers indicated that the team involved them by informing them about the process. This health service normally lasted for a couple of weeks. When home care took over responsibility for the care of the patient, the informal caregivers found that the information ceased and the information problems only were delayed to the time after the interdisciplinary team has left the scene:

*The multidisciplinary team was very good at contacting and assessing and informing, so I thought it worked very well, but now we really hear nothing more when home nursing has taken over.* (C9)

The relatives seemed to accept that the approach at the hospital was biomedically based, but they missed the follow-up provided by the hospital staff after discharge, which was tailored to the needs of elderly patients:

*But exactly at discharge … we realised that the stay was going to end because they found nothing in a way which they could explain, and we already know that patients may not be in the hospital if they are not sick and need treatment … So then we realised that, then we asked: ‘What about stay or rehabilitation in another department?’ … because she has been to another department at the hospital before, at rehab. At the hospital, there, she was treated really well. So, we wanted that. We were a little unsure of her condition, how she was going to be when she got home and what responsibilities we were given, and the neighborhood care and things like that. But then we really only got a message the day she was sent home, that now she had been sent home. And then we were kind of thinking, What do we do now, then? It was rather bad, but then we were told by the hospital that an multidisciplinary team would come from the district to my aunt’s home.* (C13)

### III: disclaimer of responsibility – the gap between the hospital, the city district and the intra-city district

The quality of the health services was viewed as a function of their ability and effort relative to contextual impediments/barriers or facilitation. Planning activities for patient transfer or discharge were often lacking, and caregivers were given little time to plan for providing assistance. After discharge, the participants found that a disclaimer of responsibility appeared. It became unclear to them who was responsible for the medical follow-up of the patient or who to contact if medical questions arose. This was particularly evident in cases where the patients were waiting for further examinations or when they had left the hospital with unresolved medical issues:

*When she (my mother) had left the hospital, they signed out/opted out/resigned. On the one hand, the patient is in a medically investigative phase, but in that medically investigative phase they sign out and leave the responsibility to the primary health service to carry out examinations that she has actually received from the hospital. I’m not sure who feels responsible for what. She is in a kind of middle layer or interlayers that I find hard to imagine.* (C5)

The responsibilities with regard to roles and coordination were perceived as unclear and not understood. The caregivers suggested that the general practitioner (GP) could be an important representative for an interdisciplinary team working in primary care, and one said that the informal caregivers should recognise that the GP is responsible for working in partnership with other agencies and organisations to ensure that the services provided are safe and of high quality:

*As her GP, he is the one who has the main responsibility, so it’s rather interesting really how much he keeps up with it.* (C8)

However, the patients’ GP rarely had experience as part of the care collaboration team:

*Me, I don’t think the GP is so informed about things. She does her job and then finishes it, somehow. There is no community. Everyone works on their own, I feel.* (C6).

The caregivers sometimes found it challenging to get full attention, particularly from the GP:

*I do not know how much the GP has been informed about her situation … the home situation … that is, I do not know.* (C8)

As older patients’ healthcare needs become more complex, they often experience challenges with managing them, and caregivers must play a major role especially, for example, for individuals with acquired hearing difficulties due to old age. One caregiver illustrated this issue:

*Yes, I tend to be … He hears badly, so I think it’s okay, and I’ve been engaged for a long time. I’ve been with Per to the doctor, because when the doctor asks, ‘How’s it going?’ he says he feels so good, but that’s not the case. So it’s rather important that I’m with him. He doesn’t really care that much. To tell the truth, I think he is tired of life. It works like that. He has been a very serious man, been high up in the lodge... He has given up. I don’t think he experiences joy anymore and does not care …* (C3)

The relatives feel that the responsibility to ensure proper healthcare rests on their shoulders:

*As family members, we feel that you should follow her up even more closely, but then it should somehow fit into everyday life and everything else you should try to do as well.* (C13)

*Yes, it would have been very nice to know something of how they look at her and how they evaluate this situation. You get a little prick of conscience, that you should follow her even more closely, but then it will somehow collapse into everyday life and everything else you should try to do too also collapses.* (C9)

### IVA: a struggle to secure professional care – the tension between competence and incompetence

Relatives did not think that the hospital is not suitable for old people, and due to the lack of sufficient information and collaboration between the services, informal caregivers indicated that the health services left them alone with minimal help. Some of them even felt forced to be the primary care coordinator for the patient. Furthermore, the caregivers realised that caring for the health of older patients requires a holistic approach in order to meet their complex needs.

One caregiver did not believe that hospitalisation contributes to better health:

*Well, I’ve noticed that when she’s been at the hospital and short time stay at the intermediate care unit, she’s been lying down, and that’s the worst thing that can happen. So, I am really saying that she should rather not go to hospital or KAD, to put it that way, but I do not know where I should have sent her to do something positive about it. She becomes passive, and the situation around her is passive. Some sort of criticism of the hospital and KAD is there, but I realised that the system is different, so I understood that they did not have time to do what I wanted them to do*. (C16)

Improved communication between caregivers and healthcare providers as well as between healthcare providers themselves is necessary to facilitate coordination of care and facilitate recovery. One caregiver expressed this sentiment:

*Everyone works on their own … and then I have to get information from everyone myself if I want to be 100 per cent updated. That means I have to join in and ask all the way. It becomes my responsibility to take responsibility for her having a good life in a way that I must take responsibility for all those agencies. So, I wish we could have a contact person, I think so*. (C6)

On top of this, the participants found that the visits from the health personnel in the primary care system were short and quick, with no time for anything other than the most important medical check on the elderly patient:

*They don’t have that many minutes, because everything is controlled by the clock*. (C11)

Most of the caregivers mentioned short visits. This resulted in little time for conversation or discussing what is important for the patient in the situation they are in now:

*Those who come here, will stay for about 4 minutes. ‘What time do you have?’ After all, I have heard that they have minutes available for every client, patient or what do you call it.* (C13)

*I realised the decision from the district said that she was granted home nursing or home care twice a day. And then they have stipulated it to be so and so many hours a week, and by doing a quick calculation, it means that there is only about 8–10 minutes for each visit.* (C9)

This made the informal caregivers ask for a more ‘*professional’* (C11) service so their loved ones could be greeted with dignity and a *‘hello, how are you today?’* (C13, C11, C1). Several of the caregivers also reported a broad use of temporary workers and personnel without medical education. This made them question the quality of the services:

*Everyone who has been here is unskilled. I think it should be a quality service; that is important. Everything you do should be quality. Many of those who have been here, they know nothing. They can’t speak Norwegian at all, and they must! They must raise their competence in this profession.* (C11)

Due to the short visits from the healthcare personnel and a service that focused on diagnosis rather than on listening to the patients’ concerns or addressing their need for practical assistance or social participation, the informal caregivers were left to help the patients with practical tasks:

*I try to come at least three times a week, maybe sometimes more. And then I drive her to the dentist and hairdresser, and all that stuff, that’s what I do. While my brother, he copes with the clothes, thankfully.* (C1)

*I take responsibility for him pretty much. I wash clothes, I do everything and shop and everything that is. Going to the pharmacy, following him all the way.* (C11)

The caregivers indicated that the patients have trouble expressing their needs and preferences with regard to the services. They felt that the patients preferred for the informal caregivers to help them meet their needs indeed:

*She trusts me to take care of her, because she is used to doing that.* (C6)

*She wants to have me there as often as possible*. (C1)

Another caregiver found there were challenges in her collaboration on personal healthcare preferences. The collaboration did not take place on the caregiver’s premises, and the representative managing intermediate care phoned her, and they had the following conversation:

*‘Can you come to a meeting at two o’clock tomorrow?’*

*‘Yes, but it’s in the middle of working hours.’*

*‘Yes, but it is very important for your grandmother, so … ’*

*‘Yes, of course, but it’s like that … ’*

*There is no such thing as ‘When can you fit it in?’*

*It was like: You have to come when it suits us. The district does not seem to be on our side, which is very frustrating. You have to try to explain your problems to them, and you almost have to exaggerate to get through to them. They just deny the issues you raise, and it is tiresome and difficult to have to deal with them*. (C23)

## Discussion

The aim of this study was to explore informal caregivers’ views on the quality of health services offered to their older relatives in the hospital and in the first 30 days following discharge. Our findings show that the informal caregivers face many challenges during this period. They reported that the healthcare system seemed to pay little attention to the mutual understanding of those involved in discharge, treatment and coordination. The participants experienced that the healthcare providers’ main focus was on the patients’ diseases, even though the services are supposed to view patients holistically, taking into consideration their values, needs, social circumstances and functional abilities. The caregivers asked for more responsiveness to each patient’s needs and preferences as well as opportunities for patients and carers to engage in the care processes. They indicated that stays in the hospital were short due to financial considerations. After discharge, the participants reported receiving insufficient information about the patients’ health condition, too little information about the organisation of the services in primary care and scant information about where to turn if they had questions concerning the patients’ health and care. They questioned who was responsible for the medical follow-up of the elderly patients, and they stated that they were left alone by the health services with the responsibility for practical tasks and securing necessary and sufficient care for the patient. Our findings indicate that the practice of engaging patients and their families in day-to-day care was not systematically incorporated into the routines of the health services. Accordingly, the engagement of older adults and their caregivers in care and care planning is essential to enhance patient and informal caregiver outcomes in transitional care [42].

Our findings have shown that translating policy into action is challenging. Rapid hospital discharge together with lack of in-home care, home-visiting services and communications with caregivers leads to difficulties for the elderly patients themselves, but also, as our participants indicated, for the informal caregivers. As the average length of stay declines, elderly people are discharged from the hospital ‘quicker and sicker’ than before [14, 16], which has implications for the informal caregivers in the current study. Our informants found that healthcare providers have less time to coordinate services across settings and to prepare the patients for the home situation [43], and this leads to informal caregivers receiving limited information about the patients’ health and care after discharge. They did not know whom to ask or who was responsible for the medical follow-up of the patient. Quality healthcare means providing care that is integrated and coordinated across levels [8], and poor communication and suboptimal care coordination [44] across care settings result in more frequent hospital readmissions and poor quality outcomes [45]. The participants found that the lack of information made the follow-up of the patients after discharge challenging. Not only did they themselves lack information about the patients’ health status and services, but they also experienced an insufficient flow of information between the different levels in the healthcare system and between different primary care services. Furthermore, they questioned the lack of focus on the patients’ preferences and needs the informants had been given a disclaimer of responsibility and were not able to grasp the organisation of the services. This made it hard for them to understand the health services’ role and what responsibility they as informal caregivers were expected to take. This corresponds well with research that reveals that older patients and their caregivers often do not feel involved in their own rehabilitation process [46, 47], and further, that staff require education and training to ensure patient participation in a way that empowers the patients [48].

Informal caregivers are essential in contributing to the care of elderly patients [49]. Nevertheless, and in line with our findings, studies have shown that older persons and their families lack participation in healthcare and care planning [48]. According to our results, there seems to be too little focus on including the informal caregivers in the information flow that goes between levels of the healthcare system when patients are being transferred as well as on supporting caregivers’ participation. Information flow is crucial to ensuring integrated care [50]. For this group of patients, the quality of healthcare services depends greatly on good planning [44, 51], and the process must include effective collaboration and communication between patients, their informal caregivers/next of kin and health professionals [18, 52]. Despite a focus in recent years on improving the coordination of care across care settings, our findings show that, to secure quality healthcare for elderly patients, more and better information must be given to informal caregivers in this process.

A lack of time was an issue we heard from our participants repeatedly, and time constraints and competing priorities are well-established barriers. The informal caregivers reported that the hospital services were concerned with examining the patients, diagnosing them and returning them back home as rapidly as possible. Despite decades of attention to the principles of activating, empowering and engaging the patients in their own healthcare [9, 53–55] as part of securing patient-centeredness as a cornerstone of healthcare service quality [54, 56], the informal caregivers reported a health service that did not appear to be responsive to the patients’ or the informal caregivers’ preferences or needs. Instead, the informal caregivers we interviewed found the health services in the municipality to be focused on short visits from healthcare personnel and the delivery of health services rather than helping with practical tasks. This focus on medical tasks can lead to the neglect of patients’ actual needs, where the medical follow-up is seen as an intrinsic good in itself rather than as a means to help individuals achieve goals that are important to them [57]. This is in line with the informants’ experiences; few reported being asked about the patients’ goals or what mattered to them or the patients. This failure to honor the patients’ goals can result in the overuse of disease-focused treatments at the end of life [57], and it illustrates services without a patient-centred focus.

To fulfil the tasks of healthcare, there must be collaboration between public services and relevant actors, such as volunteers, neighbours and relatives [58]. When assessing the health needs of older persons, it is important to consider not only the specific diseases they may be experiencing but also how these interact and affect their functioning in daily life [59]. The participants in our study described how, when the health service was unable to assist the patients for organisational and systemic reasons, they helped with practical needs. This is consistent with other studies showing that informal caregivers take responsibility for a wide range of different tasks [60]. In addition to these practical tasks, the participants indicated that they felt responsible for the patients’ security and for coordinating the services. The concept ‘burden of care’ has been discussed and acknowledged for years [61]. However, when the services leave it to the informal caregivers to secure and coordinate the health services for elderly patients, the impact on quality of care must be considered, with particular attention to the different competencies of the informal caregivers taking this responsibility. The informal caregivers stated that they, in many cases, felt responsible for securing the quality of the healthcare provided to the elderly patients. Without substantial improvements in home care services for older people, older people may often fail to receive the quality care they need [52]. Welfare states have been described as having guiding principles such as equity and equality [62]. These are also essential aspects of quality of care according to WHO [8]. As health services are increasingly transferring responsibility for the services to informal caregivers, who have variable resources and competences, we must ask ourselves whether the principles of quality care and the welfare state are at risk.

## Conclusion

Our findings indicate that informal caregivers are aware of the importance of engaging in the healthcare of elderly patients to ensure a high quality of health services in the hospital and during the critical 30-day period after discharge. To ensure good quality, our findings suggest that health services must be organised in ways that makes it possible for the healthcare personnel to take into account each person’s habits, needs and preferences when planning and delivering care for old patients. For more effective delivery of healthcare, the interdependence within and between interprofessional care teams and the persons they serve needs to be better coordinated. Healthcare professionals needs communication skills and enough time to be able to include the informal caregivers in management during transitions of care and follow-up healthcare after discharge, through designated family meetings, clinical bedside handovers, ward rounds and admission and discharge consultations. In line with other reseach, we suggest that future work needs to evaluate targeted strategies relating to family members’ contributions to managing healthcare transitions and follow-ups after discharge, with outcomes directed towards families’ understanding of these changes and their input in preventing and identifying treatment-related problems [1]. Greater attention should be placed on enabling shared decision-making during planned communication encounters involving caregivers, bedside handovers, ward rounds and admission and discharge consultations.

To achieve a high standard of healthcare for older patients during their hospital stays and in the 30-day period after discharge healthcare services needs to develop cultures and structures that facilitate informal caregivers’ and older patients’ participation in their own care. Future studies should focus on how to implement such strategies to develop the healthcare professionals’ interpersonal skills and communication with the patients and their family caregivers to ensure a more user-friendly process.

## Data Availability

The datasets generated and/or analysed during the current study are not publicly available due to privacy concerns but are available from the corresponding author upon reasonable request.

## Abbreviations

COREQ: Consolidated criteria for reporting qualitative studies
EU: European Union
GP: General Practitioner
KAD: Municipal emergency 24-h places
OECD: Organisation for Economic Co-operation and Development
REC: The Regional Committees for Medical and Health Research Ethics
WHO: World Health Organisation
